# Dietary Compound Resveratrol Is a Pan-BET Bromodomain Inhibitor

**DOI:** 10.3390/nu9111172

**Published:** 2017-10-27

**Authors:** Luiz Antonio Dutra, David Heidenreich, Gabriel Dalio Bernardes da Silva, Chung Man Chin, Stefan Knapp, Jean Leandro dos Santos

**Affiliations:** 1 School of Pharmaceutical Sciences, São Paulo State University (UNESP), Araraquara 14800903, Brazil; luizdutra_qf@yahoo.com.br (L.A.D.); gabriel.dalio@hotmail.com (G.D.B.d.S.); 2 Institute for Pharmaceutical Chemistry and Buchmann Institute for Life Sciences, Goethe-University, D-60438 Frankfurt am Main, Germany; heidenreich@pharmchem.uni-frankfurt.de (D.H); Knapp@sgc.ox.ac.uk (S.K)

**Keywords:** resveratrol, bromodomains, epigenetic, Differential Scanning Calorimetry (DSF), Isothermal Titration Calorimetry (ITC)

## Abstract

The chemopreventive and anticancer effects of resveratrol (RSV) are widely reported in the literature. Specifically, mechanisms involving epigenetic regulation are promising targets to regulate tumor development. Bromodomains act as epigenetic readers by recognizing lysine acetylation on histone tails and boosting gene expression in order to regulate tissue-specific transcription. In this work, we showed that RSV is a pan-BET inhibitor. Using Differential Scanning Fluorimetry (DSF), we showed that RSV at 100 µM increased the melting temperature (∆Tm) of BET bromodomains by around 2.0 °C. The micromolar dissociation constant (*K*_d_) range was characterized using Isothermal Titration Calorimetry (ITC). The RSV *K*_d_ value accounted to 6.6 µM in case of BRD4(1). Molecular docking proposed the binding mode of RSV against BRD4(1) mimicking the acetyl-lysine interactions. All these results suggest that RSV can also recognize epigenetic readers domains by interacting with BET bromodomains.

## 1. Introduction

Resveratrol (3,5,4′-tryhydroxy-stilbene) is a naturally occurring polyphenol found in apples, peanuts, plums, berries, and fruit seeds of *Vitis venifera* species. This compound has received worldwide attention due to its role in regulating multiple transduction pathways of several diseases, including cancer, cardiovascular, neurodegerative, and inflammatory diseases [1].

For cancer, RSV interferes in the different stages of tumors development, such as initiation, promotion, and progression [2]. The role of this compound on angiogenesis and metastasis is also described in the literature [3,4]. Both intrinsic and extrinsic apoptotic pathways are involved in apoptosis induced by RSV [5]. It has been described that the anticancer and chemopreventive effects of RSV are associated with activation/upregulation of the mitogen-activated protein kinase (MAPK) and sirtuins (SIRT). Moreover, inhibition and/or downregulation of the following targets/pathways are described for RSV: the phosphatidylinositol 3-Kinase (PI3K) pathway, cyclin-dependent kinase 4 and 6 (CDK4 and CDK6), cyclins D1 and D3, and the signal transducer and activator of transcription 3 (STAT-3), p21 and p53 [6].

Among the multiple effects of RSV against cancer, those involving the regulation of epigenetic mechanisms seem to be promising to regulate tumor development [7]. RSV is able to activate sirtuin-1 (SIRT-1), a class III of histone deacetylase (HDAC), leading to transcriptor factors deacetylation and cell proliferation inhibition [8]. In addition, this compound is a pan-HDACs inhibitor acting against eleven different HDACs of class I, II, and IV in a dose-dependent manner [9]. Despite these well-established effects, RSV activity against other epigenetic targets such as bromodomains is still unknown.

Bromodomains (BRDs) are epigenetics “readers” because they recognize lysine acetylation in histone tails and other nuclear proteins to boost gene expression, which has been involved in the development of many diseases, including cancer. Currently, 61 bromodomains present in 46 proteins have been reported [10]. Crystallographic data revealed the binding mode of acetyl-lysine (KAc) to bromodomains, showing that the acetyl group binds to the central hydrophobic pocket of BRDs, anchored by asparagine 140 residue (Asn140) and mediated by water molecules [10]. Bromo and Extra Terminal domains (BET)-BRDs comprise four members in humans (i.e. BRD2, BRD3, BRD4, and BRDT), whose dysfunction has been associated with the development of NUT midline carcinoma [10,11]. BRD4(1) has received attention after the discovery of the potent and selective pan-BET inhibitor (JQ1), which exhibited outstanding antiproliferative activity against the BRD4(1)-dependent cell line [11]. A different compound, RVX208, which is structurally related to RSV, is a bromodomain inhibitor that recognizes the second bromodomain in BETs. For this molecule, high selectivity (superior to 20) was characterized for BD2 of BRD2 and BRD3 [12]. 

In order to evaluate the ability of the dietary compound RSV to inhibit BET-BRDs, we performed here the screening against BRD2, BRD3, BRD4, and BRDT using Differential Scanning Fluorimetry (DSF). Isothermal Titration Calorimetry (ITC) was used to determine the binding constant (*K*_d_) for BRD4. Moreover, the binding mode of RSV into Kac of BRD4(1) was proposed using molecular docking.

## 2. Material and Methods

### 2.1. Protein Expression

#### 2.1.1. Low Scale Expression

Plasmid vectors coding bromodomains (BRD2(1), BRD3(1), BRD4(1), BRDT(1) and BRD4(2)) were added into Eppendorf tube (Greiner Bio One, Kremsmünster, Austria) containing chemically competent *E. coli* BL21 (DE3) cells, which were kept on ice for 30 min. For this solution, heat shock was performed at 42 °C for 40 s, and then it was kept in on ice. Luria-Bertani (LB) Broth (Carl Roth, Karlsruhe, Germany) medium was added (100 µL) and all cells were grown at 37 °C for 1 h. After, cells were selected on plates enriched by agar containing kanamycin (50 µg/mL) (Carl Roth, Karlsruhe, Germany) and chloramphenicol (34 µg/mL) (Amresco, Solon, ME, USA). Then, 50 mL of 2× LB medium concentration with kanamycin (50 µg/mL) (Carl Roth, Karlsruhe, Germany) and chloramphenicol (34 µg/mL) (Amresco, Solon, ME, USA) were inoculated with the selected cells. The cells were grown overnight (200 RPM, 37 °C).

#### 2.1.2. Large Scale Expression

Terrific broth medium (TB medium) was prepared in advance (4× concentrated and autoclaved). This medium was diluted in water (1 L) and the medium containing the cells and kanamycin (50 µg/mL) (Carl Roth, Karlsruhe, Germany) was pipetted at a volume of 10 mL. The cells were grown up to 1.5 of Density Optical (DO) (200 RPM, 37 °C). The shaker was set up at 18 °C, and the cells were grown up to 3.0 DO (180 RPM) and then induced overnight with 500 µM of Isopropyl β-D-1-thiogalactopyranoside (IPTG) (Amresco, Solon, ME, USA). After, the cells were harvested at 8.700 g using centrifuge Sorval LyNX 6000-Thermo (Thermo Fisher Scientific, Waltham, MA, USA) and stored at −20 °C.

### 2.2. Protein Purification

#### 2.2.1. Affinity Chromatography with Co^2+^ Beads

The harvested cells were resuspended in 30 mL lysis buffer (50 mM HEPES pH 7.4, 500 mM NaCl, 1 mM TCEP, 15 mM imidazole, and 5% glycerol) and lysed using a cell sonicator (35% amplitude, cycling 5 s ‘on’, and 10 s ‘off’ for 3 min). After that, 0.15% of Polyethyleneimine (PEI) (Fluka (Honeywell), Morris Plains, NJ, USA) were added and the cell debris was centrifuged (23.000 RPM, 30 min, 4 °C). Co^2+^ beads (5 mL) were added to a glass column (3 cm diameter), washed with water (50 mL) and followed by equilibration using lysis buffer (50mL). The cellular lysate containing protein was eluted through stationary phase chromatography using imidazole in lysis buffer (30 mM, 50 mM, 100 mM, and 2× 300 mM). This procedure was performed at 4 °C and purity fractions were analyzed by SDS-PAGE [13]. The fractions were concentrated by centrifugal filters (Merck Millipore, Burlington, VT, USA) and stored at 4 °C.

#### 2.2.2. Rebinding with Ni^2+^ Beads

Protein fractions were gathered from Co^2+^ beads column followed by His-tag cleavage using TEV (20× less TEV concentration than protein concentration) in 20 mL TEV buffer (30 mM HEPES, 300 mM NaCl, 0.5 mM TCEP, 5% glycerol) at 4 °C overnight. That protein solution was applied to a column containing 2 mL of Ni^2+^ beads. The stationary phase chromatography was equilibrated with TEV buffer (12 mL), and then the sample was eluted and collected completely followed by two more elutions (12 mL each) using 30 mM and 300 mM imidazole in TEV buffer, respectively. All procedures were performed at 4 °C. Those samples were analyzed by SDS-PAGE and concentrated through centrifugal filter (Merck Millipore, Burlington, VT, USA) up to 20 mg/mL and stored at 4 °C.

#### 2.2.3. Size Exclusion Chromatography (SEC)

The chromatography column Superdex 75 Increase (GE HealthCare, Little Chalfont, UK) was equilibrated beforehand using gel filtration buffer (10 mM HEPES, 150 mM NaCl, 0.5 mM TECP, and 5% glycerol) at flow of 1 mL/min. Samples were loaded through loop of 5 mL using that column previously which was connected to chromatograph ÄktPrime^TM^ plus (GE HealthCare, Little Chalfont, UK) using gel filtration buffer as mobile phase. All proteins were collected in a range volume of 75–80 mL, analyzed by SDS-PAGE, concentrated up to 10 mg/mL, and then stored at −80 °C.

### 2.3. Differential Scanning Fluorimetry (DSF)

All bromodomains were prepared at 2 µM of final concentration in 2 mL of DSF-assay buffer (10 mM HEPES pH 7.5 and 500 mM NaCl). Sypro Orange dye (Thermo Fisher Scientific, Waltham, MA, USA) (2 µL), used as fluorescence probe, was added into the sample protein, and then the samples were pipetted (20 µL) into 96-well microplates. All compounds were diluted beforehand (500 µM) in dimethyl sulfoxide (DMSO) (Carl Roth, Karlsruhe, Germany) and stored into 96-well plates at −20 °C. RSV was pipetted into protein samples (at 10 µM and 100 µM of final concentration) and plate sealed with PCR seal. Δ*T_m_* data for each compound was performed by qPCR machine (Agilent, Santa Clara, CA, USA), which had temperature raises of 3 °C per min in 71 cycles, 1 degree step, 21 s, with a starting temperature of 25 °C [11].

### 2.4. Isothermal Titration Calorimetry (ITC)

Experiments were carried out on a Nano ITC microcalorimeter (TA Instruments, New Castle, PA, USA). The experiment was carried out at 15 °C and stirring at 350 RPM in ITC buffer (10 mM HEPES pH 7.4, 150 mM NaCl, and 0.5 mM TCEP). BRD4(1) was loaded using a microsyringe (250 μL) in ITC buffer. Initial titrations had injection of 4.0 μL followed by 21 injections of 8 μL spacing among them at 200 s between injections. The collected data was processed through NanoAnalyze™ software (Version 3.5.0, TA Instruments, New Castle, PA, USA) supplied with the instrument to yield enthalpies of binding (*ΔH*) and dissociation constants (*K*_d_). Thermodynamic parameters were calculated (*ΔG* = *ΔH* − TΔS = −R*T*lnK_B_, where *ΔG*, *ΔH* and *ΔS* are the changes in free energy, enthalpy, and entropy of binding respectively). In all experiments BRD4(1) was titrated into RSV solution (reverse titration) [11].

### 2.5. Molecular Docking

Molecular modeling studies were performed with Maestro v9.1 Software (Schrödinger Inc., New York, NY, USA) [12].The crystal structure of BRD4(1) (PDB code: 3MXF) complexed with inhibitor JQ-1 was imported from Protein Data Bank (PDB) to Schrödinger Maestro suite 2016 [14] applying OPLS3 (Optimized Potential for Liquid Simulations) force field. The crystallographic and conserved water molecules in the active site were not deleted. Hydrogen atoms were added, and formal charges, along with bond orders, were assigned to the structure. RSV was prepared for docking using LigPrep (version 11, Schröndinger, New York, NY, USA). This procedure aimed to optimize the ligand geometry, to generate low energy 3D structure, and to eliminate structural mistakes in ligands using the OPLS3 force field. The ionization states of ligands was studied at pH 7.0 ± 2.0 using Epik (version 11, Schröndinger, New York, NY, USA) [15]. The grid was generated as a cubic box of 10 Å × 10 Å × 10 Å centered on the active site residues. The Standard Precision (SP) and Extra Precision (XP) flexible ligand docking were performed using Glide within Schrödinger-Maestro v9.1 [14]. The final score was obtained for energy-minimized poses and the best-docked pose with lowest glide score was selected for RSV. Docking protocol was validated by re-docking the ligand JQ-1 to protein, thereby aiming to verify the software capability of reproducing the same pose of the ligand observed in the crystal structure. The quality of this result was analyzed by Root-mean-square deviation (RMSD) between pose of original crystal structure and re-docked ligand. Distances up to 2 Å were considered reliable to this docking protocol [15].

## 3. Results

### 3.1. Differential Scanning Fluorimetry (DSF) and Isothermal Titration Calorimetry (ITC) of RSV against Bromodomains

We have employed thermal stability assay using DSF method to evaluate the ability of RSV (at 100 µM) to interact with several BRDs of the BET family, comprising the first and second bromodomains (BD1 and BD2). In the presence of RSV, the melting temperature shifts (∆T_m_) of BRDs increased at values of 2.0 °C (BRD4(1)), 2.9 °C (BRD2(1)), 1.8 °C (BRD3(1)), 1.5 °C (BRDT(1)), and 3.0 °C for BRD4(2), respectively (Table 1). These results revealed that RSV interacts with all of those BD1 BD2 bromodomains.

**Figure 1:**
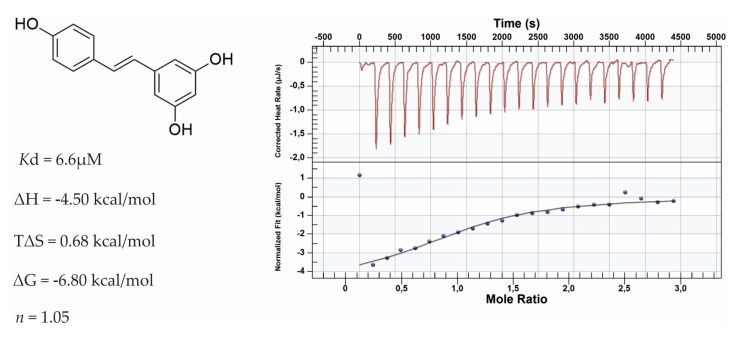
Isothermal Titatrion Calorimetry (ITC) data for RSV. Reverse titration of BRD4(1) into RSV buffered, BRD4(1): 400 µM, RSV: 17 µM.

Orthogonal assay using ITC was performed in order to confirm the data observed for DSF studies. For this assay, BRD4(1) was used due to its well established involvement in cancer development [16]. The obtained dissociation constant (*K*_d_) using ITC has demonstrated *K*_d_ value of 6.6 µM for RSV against BRD4(1) (Figure 1). The affinity of RSV for BRD4(1) was due to large negative binding enthalpy (*ΔH*) values, suggesting favorable polar interaction with this ligand. It was found that *ΔH* had values of −4.5 Kcal/mol. On the other hand, RSV appears to be revealing hydrophobic interaction not favorable due to entropic change *ΔS* > 0. It was found, for RSV, that *ΔG* values of −6.80 Kcal/mol were against BRD4(1).

### 3.2. Binding Mode of RSV into Kac Binding Site of Bromodomains

**Figure 2:**
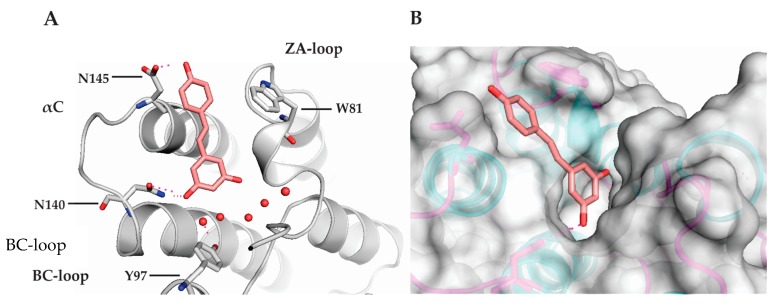
Molecular docking of BRD4(1) and RSV. **A**, hydrogens bond formed with Asn140 in the Kac binding site from BC-loop and Asn145 from αC. **B**, RSV interactions into Kac binding site and open ZA-loop pockets in surface mode. W81: Tryptofan 81; Y97: Tyrosine 97; N140: Asparagin 140; N145: Asparagine 145.

The possible binding mode of RSV against BRD4(1) and its interaction with the conserved Kac binding site of this bromodomain were characterized through docking studies. The result suggests that RSV occupies the Kac binding site with interactions mimicking hydrogens bond of acetyl-lysine [11]. In this case, interactions of RSV are limited to the 3,5-disubstituted ring, whose hydroxyl groups interact through hydrogens bond with Asn140 residue in the BC-loop. Unlike others inhibitors and acetyl-lysine crystal structures, RSV does not make a water-mediated hydrogen bond with Asn140 into Kac binding site (Figure 2A) [17]. On the other hand, the mono-substituted ring is oriented toward to the opened ZA-loop pocket, revealing polar interaction with Asn145 in αC region (Figure 2B). In agreement with ITC data measurement, the molecular docking shows limited polar interactions for RSV besides lacking hydrophobic interactions, which is confirmed with *ΔS* > 0 value. Hits optimization aiming to catch up hydrophobic interactions, such as π-stacking with Trp81 into ZA-loop region, appears to provide affinity for BRD4(1) inhibitors [18,19]. The validation of docking model showed a RMSD value of 0.65 Å.

## 4. Discussion

Modification upon chromatin structure such as methylation, acetylation, and phosphorylation have been broadly exploited as key point of understanding epigenetic mechanisms [20]. Lysine acetylation upon histone tails made by histone-acetyl transferases (HAT) drives to better accessibility of transcriptionally active genes to chromatin structure [21]. BET-BRDs recognize histone acetylation (“readers”) and regulate gene expression involved in many diseases as cancers, inflammation, neurological disorders, and cardiovascular [20].

In this work, we demonstrated the capacity of resveratrol to interact with BET-BRDs using biophysical methods besides suggesting its binding mode into Kac of BRD4(1) through molecular docking studies. Therefore, we performed screening of RSV against BET-BRDs (BRD2, BRD3, BRD4, and BRDT) by setting DSF and ITC experiments. RSV showed thermal shift value for all BET-BRDs which ranged from 1.5 to 3.0 °C. ITC experiments confirmed the ability of RSV (*K*_d_ = 6.6 µM) to interact with BRD4(1). The molecular docking study allows for the proposing of the mode of interaction of RSV with BRD4(1). Our data showed that RSV has a similar binding mode into Kac of BRD4(1) compared to other BRDs-inhibitors, which mimics polar interaction of acetyl-lysine with Asn140 into Kac [11,12]. Unlike others BRDs-inhibitors, RSV lacks hydrophobic interaction due to *ΔS* > 0 value. Hydrophobic interactions such as π-stacking with Trp81 into ZA-loop region appear to provide a higher affinity of inhibitors for BRD4(1) [18,19].

BRD4 has been reported as target in various cancer types as ovarian, breast, gastric, andregulation of transcriptions factor MYC [6]. Interestingly, several BET inhibitors are on clinical trials targeting many cancer types, including the compounds OTX015 and GSK525762 for NUT midline carcinoma, acute myeloid leukemia and myelodysplastic syndrome (NCT02308761), and lymphoma (NCT01949883) and multiple myeloma (NCT02157636) [22]. Besides the activation of SIRT-1, which leads to deacetylation of transcription factors [23], RSV could present additional anticancer effect through BRD4 inhibition. SIRT-1 activation was demonstrated to suppress mammalian target of rapamycin (mTOR) in HeLa cells (Henrietta Lacks cells). This effect drives to autophagy activation and inhibition of cancer cells proliferation [24]. SIRT-1 also increases deacetylation on histone in the autophagy promoter region, promoting the dissociation of BRD4 from gene promoters [25].

Herein, we hypothesized that the anticancer effects of RSV could be related to dual activity, including SIRT-1 activation and BRDs inhibition [25]. However, further studies are required in order to elucidate the relation between this dual activity and autophagy mechanism.

## 5. Conclusions

Resveratrol was described for the first time to interact with BET-BRDs, being classified as a pan-BET inhibitor. This interesting effect can contribute to and explain the chemopreventive and anticancer effect of this polyphenol.

## Figures and Tables

**Table 1:** Thermal stability data for bromodomains in the presence of resveratrol (RSV).

Protein	Resveratrol 100 µM
	∆T_m_ (°C)
BRD2(1)	2.0 ± 0.5
BRD3(1)	1.8 ± 0.2
BRD4(1)	2.0 ± 0.6
BRDT(1)	1.5 ± 0.3
BRD4(2)	3.0 ± 0.5

BRD: Bromodomain; BRDT: Bromodomain Testis-Specific Protein.
